# Prevalence and predictors of peripheral neuropathy after breast cancer treatment

**DOI:** 10.1002/cam4.4202

**Published:** 2021-08-14

**Authors:** Mandana Kamgar, Mark K. Greenwald, Hadeel Assad, Theresa A. Hastert, Eric M. McLaughlin, Kerryn W Reding, Electra D. Paskett, Jennifer W. Bea, Aladdin H. Shadyab, Marian L. Neuhouser, Rami Nassir, Tracy E. Crane, Kalyan Sreeram, Michael S. Simon

**Affiliations:** ^1^ Medical College of Wisconsin Milwaukee Wisconsin USA; ^2^ Barbara Ann Karmanos Cancer Institute Wayne State University Detroit Michigan USA; ^3^ The Ohio State University Columbus Ohio USA; ^4^ University of Washington Seattle Washington USA; ^5^ The University of Arizona Cancer Center Tucson Arizona USA; ^6^ University of California San Diego San Diego, La Jolla California USA; ^7^ Fred Hutchinson Cancer Research Center Seattle Washington USA; ^8^ Umm Al‐Qura’s University Mecca Saudi Arabia; ^9^ Ascension St Vincent Hospital Indianapolis Indiana USA

**Keywords:** breast cancer, cancer survivors, chemotherapy, Peripheral neuropathy, taxane

## Abstract

**Background:**

Many of the 3.8 million breast cancer survivors in the United States experience long‐term side effects of cancer therapy including peripheral neuropathy (PN). We assessed the prevalence and predictors of PN among women with breast cancer followed in the Women's Health Initiative's Life and Longevity After Cancer survivorship cohort.

**Methods:**

The study population included 2420 women with local (79%) or regional (21%) stage disease. Presence of PN was based on the reports of “nerve problems and/or tingling sensations” after treatment and PN severity was assessed using the Functional Assessment of Cancer Therapy‐Gynecologic Oncology Group/Neurotoxicity instrument. Logistic regression analysis was used to evaluate the socio‐demographic and clinical factors associated with PN prevalence and severity.

**Results:**

Initial breast cancer treatment included surgery‐only (21%), surgery and radiation (53%), or surgery and chemotherapy (±radiation) (26%). Overall, 17% of women reported PN occurring within days (30%), months (46%), or years (24%) after treatment and 74% reported ongoing symptoms at a median of 6.5 years since diagnosis. PN was reported by a larger proportion of chemotherapy recipients (33%) compared to those who had surgery alone (12%) or surgery+radiation (11%) (*p *< 0.0001). PN was reported more commonly by women treated with paclitaxel (52%) and docetaxel (39%), versus other chemotherapy (17%) (*p *< 0.0001). In multivariable analyses, treatment type (chemotherapy vs. none; OR, 95% CI: 3.31, 2.4–4.6), chemotherapy type (taxane vs. no‐taxane; 4.74, 3.1–7.3), and taxane type (paclitaxel vs. docetaxel; 1.59, 1.0–2.5) were associated with higher odds of PN.

**Conclusion:**

PN is an important long‐term consequence of taxane‐based chemotherapy in breast cancer survivors.

## 1 INTRODUCTION

Breast cancer is the most common cancer among women in the United States and second leading cause of cancer‐related death.^1^ Improvements in early detection and treatment have resulted in increased survival with current estimates of >3.8 million breast cancer survivors in the United States.^2^ Many cancer survivors suffer long‐term side effects of chemotherapy including peripheral neuropathy (PN),^3^, ^4^ which adversely impacts the quality of life.^5^

Chemotherapy‐induced PN is a common and devastating side effect of many chemotherapy agents including taxanes, platinum compounds, and vinca alkaloids.^6^, ^7^ Damage to peripheral nerves results in symptoms including tingling, burning, numbness, weakness, pain, and cramps. In some cases, chemotherapy‐induced PN is mild and reversible, whereas in others it is severe and irreversible, interfering with daily activities. In a systematic review on chemotherapy for early‐stage breast cancer, the reported prevalence of PN from 1 to 3 years post‐diagnosis ranged between 11% and 80%.^3^ In the NSABP‐B‐30 trial of lymph node‐positive, early‐stage breast cancer, participants were randomized to sequential doxorubicin, cyclophosphamide followed by paclitaxel, versus all three agents together, or concurrent doxorubicin and paclitaxel. Overall, 41.9% of participants reported PN at 2 years post‐chemotherapy, which corresponded to reports of lower quality of life.^8^ Age, type of taxane (paclitaxel > docetaxel), and history of diabetes were important predictors of PN in that trial.^9^

There are limited published data on prevalence and predictors of PN in long‐term breast cancer survivors: information is generally restricted to clinical trial participants,^8^, ^10^, ^11^, ^12^ which over‐represents younger and healthier patients,^13^ or participants from small cross‐sectional or cohort studies.^14^, ^15^, ^16^, ^17^ In the current analysis, we evaluated both the prevalence and potential clinical predictors of PN in a sample of long‐term breast cancer survivors who participated in the Women's Health Initiative (WHI) Life and Longevity After Cancer (LILAC) study. We hypothesized that PN is a chronic medical issue facing cancer survivors and that certain patient subgroups would be at higher risk. The LILAC cohort affords a unique opportunity to report on PN in breast cancer survivors from 40 centers across the United States at a median of 6.5 years post‐diagnosis.

## 2 PATIENTS AND METHODS

### 2.1 Study design

Details of the WHI design and implementation have been previously published.^18^, ^19^ Briefly, the WHI included 68,132 post‐menopausal women randomized into one or more clinical trials (CT; dietary modification, hormone therapy, and calcium/vitamin D), and 93,676 women enrolled into a parallel observational study (OS). The LILAC infrastructure cohort is a cancer survivorship cohort of WHI participants (CT and OS) who enrolled between 2013 and 2017, and who developed one of eight designated cancers after WHI enrollment.^20^

**FIGURE 1:**
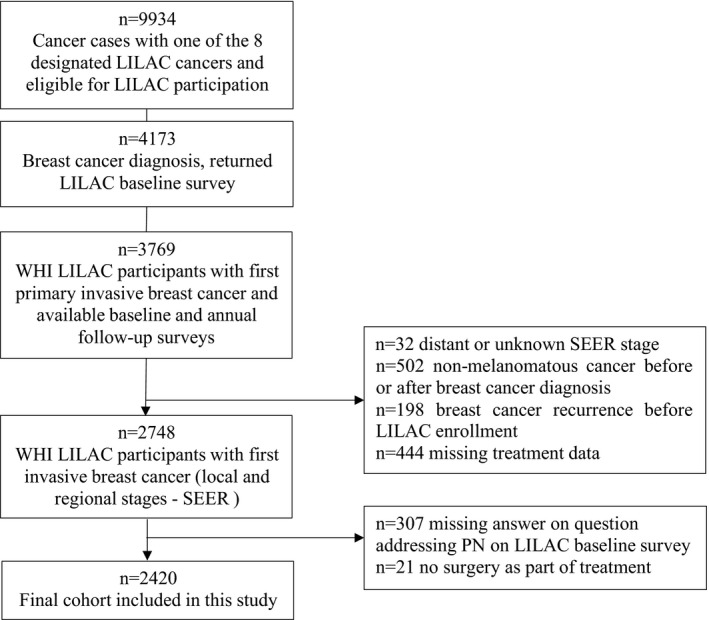
Inclusion and Exclusion Criteria (CONSORT Diagram). Abbreviations*: n*, number; LILAC, Life and Longevity After Cancer; WHI, Women's Health Initiative; SEER, Surveillance, Epidemiology, and End Results; PN, peripheral neuropathy

Of the entire LILAC cohort of 9934 women, 3769 were diagnosed with a first primary invasive breast cancer between 1994 and 2016 and completed the two required LILAC data collection instruments (surveys). Women were excluded from the current analysis if they had unknown or distant‐stage disease (*n* = 32), diagnosis of another primary non‐melanomatous cancer either before or after diagnosis of breast cancer (*n* = 502), breast cancer recurrence (either local or distant) prior to LILAC enrollment (*n* = 198), missing information on cancer treatment (*n* = 444), and missing information on PN (*n* = 307), leaving an analytic cohort of 2420 (Figure 1).

### 2.2 Assessment of peripheral neuropathy prevalence

The first or baseline LILAC survey included questions on the initial course of cancer treatment including type of treatment and start date, and symptoms experienced by the cancer patients occurring either before or after cancer treatment as well as those occurring at the time of the survey.^20^ The median time from cancer diagnosis to completion of the baseline survey for participants in the current analysis was 6.5 years (interquartile range, IQR 2.7–10.9). The survey symptom inventory included a four‐part series of questions inquiring specifically about PN; to elicit information representing what is clinically felt to be PN; the questions in the survey on PN focused specifically on “nerve problems” or “tingling sensations.” Participants were asked whether these symptoms were new to them after cancer treatment and were not due to some other medical condition. They were also asked whether these symptoms developed days, months, or years after their cancer treatment. Other questions in the survey addressed whether symptoms related to PN were still present when the participant completed the survey and whether they had received any specific treatment for their PN symptoms (Table S1).

### 2.3 Assessment of peripheral neuropathy severity

Starting at 1 year after enrollment, annual follow‐up surveys were sent to LILAC participants which included multiple measures regarding cancer survivorship including detailed assessment of PN severity.^20^ Median time from diagnosis to completion of the first follow‐up survey was 7.7 years (IQR 3.9–12.2). PN severity was assessed through the “FACT‐GOG/NTX (Functional Assessment of Cancer Therapy‐Gynecologic Oncology Group/Neurotoxicity)” (v.4) subscale, an 11‐item validated instrument.^21^ Item scores (from 0 [not at all] to 4 [very much]) were summed to yield a composite score for PN severity of 0–44; higher scores indicate higher severity. While the original FACT‐GOG/NTX was developed to assess symptoms within the prior 7 days of survey completion, participants in LILAC were asked about symptoms that occurred in the prior 4 weeks (Table S2). For this analysis, we stratified PN severity scores into tertiles including low (<5), medium,^5^, ^6^, ^7^, ^8^, ^9^ and high (10+) severity. For participants who only answered between 6 and 10 out of 11 questions, scores were prorated to the scale of 0–44 points based on non‐missing responses.^22^ There were 2187 women (90.4%) who provided complete data on PN severity: 150 (6.2%) who responded to only 10 items and 42 (1.7%) who responded to 6–9 items were included in the analysis, and 41 (1.7%) who responded to ≤5 questions were excluded from the severity analysis.

### 2.4 Cancer treatment

Data on the initial course of cancer treatment (surgery, radiation, and chemotherapy) including type and timing of treatment, were derived from Medicare linkage for women age ≥65 at diagnosis (*N* = 823, 34%), or through direct medical record abstraction if diagnosed since 2000 and not covered by Medicare (*n* = 1597, 66%). Medical record abstraction was used as the source of information on treatment when both data sources were available.

### 2.5 Demographics and baseline characteristics

Information on race, ethnicity, marital status, tobacco, alcohol, blood pressure, waist circumference (WC), body mass index (BMI), and history of diabetes and osteoarthritis were derived at WHI enrollment. Blood pressure, WC, and BMI were measured annually for the WHI CT participants and at baseline and year 3 for those in the OS. Clinical values used for analysis were those collected closest to and prior to the date of breast cancer diagnosis (median years from data collection to diagnosis: 5.8 years). Baseline WHI data were used when follow‐up information was missing. A fully conditional specification imputation model was used to stochastically impute the remaining missing data due to the non‐monotone missing data pattern [highest percentage of missing data: smoking status (1.6%), alcohol use (0.7%), and education level (0.6%)]. Due to the low percentage of missing data, no sensitivity analysis was performed. The imputation methodology used study arm, race, education, geographic region, marital status, smoking, alcohol, diabetes, thyroid disease, osteoarthritis, stage, BMI, WC, blood pressure, and diagnosis age.^23^

### 2.6 Statistical methods

Inverse probability weighting was used to minimize selection bias related to women who enrolled in LILAC, versus women who did not enroll but would have met inclusion criteria (*n* = 641).^24^ Weights were calculated from a logistic regression model using the same variables included in the imputation model. For univariate analyses, demographic and clinical characteristics were compared by presence or absence of PN derived from the first LILAC survey, using chi‐square tests and Wilcoxon rank‐sum tests, due to non‐normal distributions of continuous variables as determined using Kolmogorov–Smirnov tests.

For multivariable analyses, logistic regression with risk factor modeling was used. The initial model assessed the effect of treatment type (surgery alone, surgery+radiation, surgery+chemotherapy+/‐radiation) on the development of PN. A second model assessed the effect of chemotherapy type (taxane vs. non‐taxane) among women who received chemotherapy (*n* = 629). Lastly, among the 344 women who received only one type of taxane during chemotherapy, a third model compared the effect of paclitaxel versus docetaxel on the development of PN. Potential confounders listed in Table 1, as well as the time from diagnosis to completion of the LILAC survey, were added to the models if they changed the odds ratio for the primary risk factor by ≥5%. Ordinal logistic regression models were used to assess whether treatment type, chemotherapy type, or taxane type was associated with PN severity tertiles, and to examine PN severity based on timing of the reported first occurrence of PN after treatment (days, months, or years). *p‐*values reported for covariates in the multivariable models were from likelihood ratio tests. All analyses were performed using SAS 9.4 (SAS Institute Inc.).

**TABLE 1:** Relationships between peripheral neuropathy and baseline demographics, treatment, and clinical characteristics

	Nerve problems or tingling sensations		
	No (*n* = 2019)	Yes (*n* = 401)	
Variable	Median [IQR]	Median [IQR]	*p*‐value
Age at WHI screening (years)	60 [56–65]	59 [55–63]	0.0003
Age at diagnosis (years)	71 [67–76]	70 [66–75]	0.0012
BMI (kg/m^2^)	26.9 [23.7–30.8]	28.2 [24.4–32.6]	0.0005
Waist circumference (cm)	84 [76–94]	85 [77–96]	0.021
Systolic blood pressure (mmHg)	122 [111–132]	121 [111–132]	0.62
Diastolic blood pressure (mmHg)	72 [67–80]	72 [67–78]	0.72
	*N* (%)	*N* (%)	
Study arm			
Clinical trial (CT)	924 (84)	179 (16)	0.68
Observational study (OS)	1095 (83)	222 (17)	
Region			
Northeast	480 (85)	84 (15)	0.29
South	437 (82)	96 (18)	
Midwest	487 (85)	87 (15)	
West	615 (82)	134 (18)	
Education			
None/some high school	27 (84)	<11	0.41
High school diploma/GED	255 (87)	39 (13)	
Some college/associate degree	648 (82)	138 (18)	
College graduate	1078 (83)	216 (17)	
Race			
White	1843 (84)	350 (16)	<0.0001
Black	67 (68)	32 (32)	
Other^a^	106 (85)	19 (15)	
Smoking			
Never	966 (82)	214 (18)	0.09
Former	927 (85)	160 (15)	
Current	96 (83)	19 (17)	
Alcohol use			
Never	134 (80)	33 (20)	0.14
Former	268 (80)	65 (20)	
Current	1600 (84)	302 (16)	
Married			
Yes	1409 (83)	281 (17)	0.93
No	603 (84)	119 (16)	
Treatment			
Surgery alone	458 (88)	60 (12)	<0.0001
Surgery+radiation	1137 (89)	136 (11)	
Surgery+chemotherapy±radiation	424 (67)	205 (33)	
Chemotherapy regimen			
Paclitaxel containing	67 (48)	74 (52)	<0.0001
Docetaxel containing	123 (61)	80 (39)	
Both paclitaxel/docetaxel	<11	<11	
Received neither	230 (83)	47 (17)	
SEER stage			
Localized	1653 (86)	260 (14)	<0.0001
Regional	366 (72)	141 (28)	
Diabetes			
Yes	146 (83)	29 (17)	1.0
No	1873 (83)	372 (17)	
Arthritis			
Yes	743 (80)	181 (20)	0.0014
No	1273 (85)	218 (15)	
Thyroid problems			
Yes	<11	<11	0.43
No	2010 (83)	398 (17)	

Missing data: education 0.6%, race 0.1%, smoking status 1.6%, alcohol use 0.7%, marital status 0.3%, BMI 0.2%, waist circumference 0.2%, systolic blood pressure 0.04%, and arthritis 0.2%.

Abbreviations: BMI, body mass index; cm, centimeter; GED, General Educational Development; kg/m^2^, kilogram per meter squared; mmHg, millimeters of mercury; *n*, number; WHI, Women's Health Initiative.

^a^ Other: American Indian or Alaskan Native, Asian or Pacific Islander, Hispanic/Latino, or other.

## 3 RESULTS

Table 1 shows the relationships between baseline characteristics and report of PN. Of 401 (17%) who reported PN symptoms, 30% had onset within days, 46% within months, and 24% within years of treatment, of whom 25% reported receiving the treatment for PN. Of those with PN, 74% reported continued symptoms at the time of the baseline survey (median time from diagnosis: 6.5 years). Cancer treatment received included 518 (21%) with surgery alone, 1273 (53%) with surgery+radiation, and 629 (26%) with surgery+chemotherapy+/–radiation. Among those who had chemotherapy, 344 were treated with a regimen including either docetaxel (59%) or paclitaxel (41%). In univariate analysis, women were more likely to report PN if they were younger at enrollment (*p* = 0.003) or diagnosis (*p* = 0.021) and self‐identified as Black (*p* < 0.001). Other factors associated with PN included receipt of chemotherapy (33%) versus surgery alone (12%) or surgery+radiation (11%) (*p* < 0.001), and receipt of a taxane‐based regimen (paclitaxel (52%) or docetaxel (39%) versus other chemotherapy (17%), *p* < 0.001). Additional clinical variables associated with a report of PN included: more advanced stage (*p* < 0.001), obesity (*p* = 0.005), and osteoarthritis (*p* = 0.0014).

In a multivariable model after adjusting for cancer stage, receipt of chemotherapy was associated with 3.31‐fold higher odds of PN (95% CI 2.38–4.61) compared to surgery alone (Table 2). After adjusting for time from diagnosis to survey completion, women treated with a taxane versus other chemotherapy had 4.47‐fold higher odds of PN (95% CI 3.07–7.32) and in a model adjusting for time from diagnosis and BMI, treatment with paclitaxel versus docetaxel was associated with 1.59‐fold higher odds of PN (95% CI 1.10–2.50). In the latter model, each 5‐kg/m^2^ increase in BMI resulted in a 1.49‐fold increase in odds of PN (95% CI 1.03–2.14).

**TABLE 2:** Multivariable models of the relationships between cancer treatment, chemotherapy type, and type of taxane on development of peripheral neuropathy

Variable	*N*	Level	Odds ratio (95% CI)	*p*‐value
Primary exposure: Treatment type				
Treatment type	518	Surgery alone	Ref	<0.0001
	1273	Surgery+radiation	0.91 (0.66–1.25)	
	629	Surgery+chemo±radiation	3.31 (2.38–4.61)	
SEER stage	507	Regional	Ref	0.06
	1913	Localized	0.78 (0.60–1.01)	
Primary exposure: Chemotherapy type				
Chemotherapy type	277	No taxane	Ref	<0.0001
	352	Taxane	4.74 (3.07–7.32)	
Time from diagnosis to completion of the survey	152	<5 years	Ref	0.21
	186	5–9 years	1.03 (0.66–1.63)	
	291	10+ years	1.47 (0.91–2.37)	
Primary exposure: Taxane type				
Taxane type	203	Docetaxel	Ref	0.45
	141	Paclitaxel	1.59 (1.01–2.50)	
Time from diagnosis to completion of the survey	122	<5 years	Ref	0.34
	135	5–9 years	0.99 (0.60–1.63)	
	87	10+ years	1.47 (0.82–2.63)	
BMI (kg/m^2^)		5 kg/m^2^ increase	1.49 (1.03–2.14)	0.032

Abbreviations: BMI, body mass index; Chemo, chemotherapy; kg/m^2^, kilogram per meter squared; LILAC, Life and Longevity After Cancer; N, number; Ref, reference group; SEER, Surveillance, Epidemiology, and End Results; WHI, Women's Health Initiative.

Lastly, in an assessment of predictors of PN severity, in a multivariable model adjusting for stage, BMI, age, and time from diagnosis, women treated with chemotherapy versus surgery, had a 1.31‐fold higher odds of having more severe PN (95% CI 1.03–1.67) (Table 3). Other variables associated with higher severity included BMI, older age at diagnosis, and longer time since diagnosis, however, type of chemotherapy was not associated with severity (OR 1.03, 95% CI 0.72–1.48, *p* = 0.85). In women who received either paclitaxel or docetaxel, paclitaxel was marginally associated with increased PN severity (OR 1.47, 95% CI 0.96–2.27, *p* = 0.08), however, these results were not significant. The timing of PN symptoms after treatment had no impact on PN severity (*p* = 0.48).

**TABLE 3:** Severity of peripheral neuropathy in relation to cancer treatment type

Variable	*N*	Level	Odds ratio (95% CI)	*p*‐value
Univariate model				
Treatment type	507	Surgery alone	Ref	0.002
	1256	Surgery+radiation	0.77 (0.64–0.93)	
	616	Surgery+chemo (±radiation)	1.03 (0.83–1.27)	
Multivariable adjusted model				
Treatment type	507	Surgery alone	Ref	<0.0001
	1256	Surgery+radiation	0.83 (0.69–1.01)	
	616	Surgery+chemo (±radiation)	1.31 (1.03–1.67)	
Cancer stage	493	Regional	Ref	0.85
	1886	Localized	1.02 (0.84–1.24)	
BMI (kg/m^2^)		5‐kg/m^2^ increase	1.37 (1.28–1.46)	<0.0001
Age at diagnosis (years)		5‐year increase	1.31 (1.22–1.40)	<0.0001
Time from diagnosis to completion of the survey		<5 years	Ref	0.0004
		5–9 years	1.05 (0.87–1.27)	
		10+ years	1.49 (1.20–1.85)	

Abbreviations: BMI, body mass index; Chemo, chemotherapy; kg/m^2^, kilogram per meter squared; LILAC, Life and Longevity After Cancer; N, number; Ref, reference group; SEER, Surveillance, Epidemiology, and End Results; WHI, Women's Health Initiative.

## 4 DISCUSSION

The prevalence of reported PN was 17% in a large cohort of postmenopausal breast cancer survivors among whom almost 75% indicated persistence of symptoms at a median of 6.5 years post‐diagnosis. As expected, PN was strongly associated with receipt of chemotherapy, with the highest rates associated with taxanes, and with rates higher for paclitaxel than for docetaxel. In a systematic review of early breast cancer survivors, prevalence of PN ranged from 11% to 80%, >1 and <3.3 years after diagnosis.^3^ A cross‐sectional study of 296 women with ER‐positive early‐stage breast cancer treated with a taxane‐based regimen reported 58.4% with PN at a mean of 6.3 years post‐diagnosis.^16^ Others reported similar findings including an analysis of 512 women age >50 years (78% with breast cancer) who received a combination of platinum and paclitaxel‐based regimens, where 47% reported persistent symptoms after an average of 6 years post‐diagnosis.^25^ Lastly, in a cross‐sectional study of 605 survivors PN was reported at a rate of 25%–30% at ≥6 years from therapy.^4^ Variation in PN prevalence in research literature is likely due to differences in study populations, treatment, timing of assessment, patient versus physician‐reported outcomes, and evaluation instruments, however, across all studies reported PN appears to be a relatively common and persistent side effect after cancer treatment.

Similar to our findings, other researchers have reported higher rates of PN associated with taxanes^4^, ^14^, ^26^ and higher rates associated with paclitaxel versus docetaxel.^27^, ^28^, ^29^, ^30^, ^31^ In a cross‐sectional study of 605 breast cancer survivors, prevalence of PN was 15% among those who had no chemotherapy and 19%, 28%, and 43% among those with non‐taxane, docetaxel‐containing, and paclitaxel‐based regimens, respectively.^4^ Of note the prevalence of PN among 15% of women who did not receive chemotherapy in this report is similar to the 12% and 11% of our cohort who had PN symptoms whether they had surgery alone or surgery+radiation and likely represents PN related to other common co‐morbid conditions such as DM, thyroid disease, and others.

Due to lack of detailed treatment information, we could not evaluate the effects of specific combinations of chemotherapy or timing of treatment on PN. In NSABP‐B‐30, PN was highest at 24 months post‐adjuvant therapy among recipients of sequential doxorubicin, cyclophosphamide followed by paclitaxel (50%), versus all three agents together (41%), or concurrent doxorubicin and paclitaxel (35%).^8^ The NSABP trial also showed that timing of PN assessment is important: for women on the sequential treatment arm, PN prevalence was 16% prior to initiation of treatment, 19% on cycle 4 day 1, 68% by 6 months, 56% by 12 months, and 50% by 24 months.^8^ This is consistent with results of other early‐stage breast cancer trials showing high variability of PN in the first year post‐treatment,^10^, ^15^ with different temporal patterns of symptom evolution and resolution depending on regimen.^11^, ^32^ In our cohort, nearly 75% of women who reported PN at any time had persistent symptoms an average of 6.5 years after diagnosis; however, we were unable to assess the evolution of symptoms or rate of resolution over time given the lack of information on PN pre‐treatment and post‐treatment intervals. Furthermore, times from diagnosis to completing the baseline and follow‐up LILAC surveys were statistically associated with some outcomes suggesting an increase in PN severity with longer duration since treatment (Table 3); however, due to diverse factors associated with such timing, we could not determine the clinical relevance of this association.

In oncology practice, PN severity is assessed by patient‐reported outcome (PRO)^33^, ^34^, ^35^ or clinician‐reported outcome (CRO) measures.^36^, ^37^ The FACT/GOG‐NTX^21^ and EORTC‐CIPN20^34^ are commonly used for PRO, and CTCAE^38^ is commonly used in clinical trials; however, there is no gold standard for evaluating PN severity in breast cancer survivors. Multiple studies have compared PRO to CRO showing moderate to low agreement, with clinicians tending to under‐report PN severity.^27^, ^32^, ^39^, ^40^ Given that our analysis reports information from a patient survey which is roughly equivalent to PRO, it is likely that our results more accurately represent the patient experience of PN, than what might be derived from CRO as part of a randomized controlled trial. More work is needed to determine how to translate results based on FACT/GOG/NTX scores into a clinical classification schema comparable to CTCAE, preferably including rigorous objective measurements.

As shown by others,^4^, ^17^, ^31^ our results suggest that higher BMI is an important predictor of taxane‐induced PN and PN severity, and that older age is an important predictor of PN severity.^15^, ^19^, ^31^, ^41^ In our analysis, diabetes was not an independent risk factor for PN. While baseline (pre‐chemotherapy) PN is an independent risk factor for the development and persistence of PN,^4^, ^8^ and poorly controlled DM is associated with DM‐induced sensory PN,^42^ there is some discrepancy in the literature regarding the role of diabetes as an independent risk factor for the development and persistence of chemotherapy‐induced PN.^4^, ^9^, ^43^ Due to the low prevalence of diabetes in the WHI (7%), lack of consistent data on the quality of diabetes control, and lack of data on prevalence of PN prior to treatment that could be related to diabetes, our study cannot completely address the association of diabetes with new PN following chemotherapy.

Strengths of this analysis include long follow‐up, diverse study population, and use of a multi‐part survey item to address the presence of PN after treatment and a standardized and reliable instrument to assess the severity of PN. Considering that the LILAC cohort reports on PN in breast cancer survivors who were enrolled from 40 centers across the United States, we believe that the results presented from LILAC will be more generalizable to the general breast cancer population than results as presented from randomized controlled treatment trials. Limitations include possible recall bias and that PN was only assessed by a single question. While FACT/GOG‐NTX is a validated instrument to assess PN severity, it does not tie symptoms or timing of symptoms to cancer treatment, which would be problematic in our cohort without available data on PN at baseline or during treatment. We, therefore, believe it was more precise to base our initial assessment of PN prevalence on the specific question dedicated to PN in the LILAC baseline survey rather than a FACT/GOG‐NTX severity cut‐off score. We feel, however, that the question used to assess PN in first or baseline LILAC survey corresponds to how the question is asked in clinical practice. Another potential limitation includes selection bias, as LILAC participants were longer term cancer survivors than other women in the WHI who died from their cancer, and were more likely to be healthier than others who did not participate; however, this bias was minimized using inverse probability weighting as described in the methods section. Lastly, some baseline clinical characteristics such as BMI, WC, and blood pressure were measured at WHI entry, a median of 5.8 years prior to cancer diagnosis, which could limit the impact of those variables on the development of PN.

In summary, our results demonstrate a high burden of long‐term PN in a large cohort of breast cancer survivors, with a pronounced impact of taxanes on PN prevalence. These results can potentially guide decision‐making regarding treatment choice for women with early‐stage breast cancer.

## CONFLICT OF INTEREST

Mandana Kamgar, Theresa A. Hastert, Eric M. Mclaughlin, Kerryn W. Reding, Marian L. Neuhouser, Rami Nassir, Tracey E. Crane, Kalyan Sreeram, and Michael S. Simon: No conflict. Mark K. Greenwald: Honoraria––Indivior; Consulting/Advisory Role–Indivior, Supernus Pharmaceuticals, Nirsum Labs; Speakers’ Bureau––Indivior. Hadeel Assad: Consulting/Advisory Role––Daiichi Sankyo. Electra D. Paskett: Stock & other Ownership Interests––Meridian Bioscience Inc; Pfizer. Jennifer W. Bea: Consulting/Advisory Role––Women's Health Initiative; Research Funding––Disarm Therapeutics. Aladdin H. Shadyab: Employment––IBH Population Health Solutions; Consulting/Advisory Role––Rancho Biosciences.

## AUTHOR CONTRIBUTIONS

Mandana Kamgar, Hadeel Assad, Theresa A. Hastert, Kerryn W. Reding, and Michael S. Simon: Contributed to conceptualization, methodology, and project administration. Eric M. Mclaughlin: Contributed to data curation, formal analysis, and visualization/data presentation. All authors: Contributed to writing original draft, review, and editing drafts.

## ETHICAL APPROVAL

The WHI project was reviewed and approved by the Fred Hutchinson Cancer Research Center (Fred Hutch) IRB in accordance with the US Department of Health and Human Services regulations at 45 CFR 46 (approval number: IR# 3467‐EXT). Participants provided written informed consent to participate. Additional consent to review medical records was obtained through signed written consent. Fred Hutch has an approved FWA on file with the Office for Human Research Protections (OHRP) under assurance number 0001920.

## Supporting information

Table S1‐S2

## Data Availability

Women's Health Initiative data are only made available to investigators with approved proposals.
