# Supplementary material for: Prevalence and predictors of peripheral neuropathy after breast cancer treatment
Source: Cancer Med. 2021 Aug 14;10(19):6666–76. doi: 10.1002/cam4.4202 (PMC8495292; doi:10.1002/cam4.4202)
Supplement: Supplementary file 1 — Table S1‐S2 [file CAM4-10-6666-s001.docx]

Supplementary Material

**Table S1: Components of the section addressing peripheral neuropathy on baseline LILAC survey**

| Please indicate the symptoms that were **NEW** to you after your cancer treatment,  and not due to some other known medical condition. | | **If yes**, how soon after treatment did symptoms occur -- in a matter of days, months, or years? | Do you still have the symptom? | Have you been treated for the symptom? |
| --- | --- | --- | --- | --- |
| Did this occur? | No Yes^1^ | Days Months Years^2^ | No Yes^3^ | No Yes |
| Nerve problems tingling sensations |  |  |  |  |

^1^ Answer to this question was used to define prevalence of PN after cancer treatment

^2^ Answer to this question was used to define timing of onset of PN after cancer treatment (days, months or years)

^3^ Answer to this question was used to define persistence of PN at the time of completion of baseline LILAC survey

**Table S2:** **Components of the section on LILAC annual survey addressing severity of peripheral neuropathy**

| 8 **In the past 4 weeks,** how true have the following statement been for you? | | | | | |
| --- | --- | --- | --- | --- | --- |
|  | Not  at all | A little bit | Somewhat | Quite  a bit | Very much |
| 8.1 I have numbness or tingling in my hands. | 0 | 1 | 2 | 3 | 4 |
| 8.2 I have numbness of tingling in my feet. | 0 | 1 | 2 | 3 | 4 |
| 8.3 I feel discomfort in my hands. | 0 | 1 | 2 | 3 | 4 |
| 8.4 I feel discomfort in my feet. | 0 | 1 | 2 | 3 | 4 |
| 8.5 I have joint pain or muscle cramps. | 0 | 1 | 2 | 3 | 4 |
| 8.6 I feel weak all over. | 0 | 1 | 2 | 3 | 4 |
| 8.7 I have trouble hearing. | 0 | 1 | 2 | 3 | 4 |
| 8.8 I get a ringing or buzzing in my ears. | 0 | 1 | 2 | 3 | 4 |
| 8.9 I have trouble walking. | 0 | 1 | 2 | 3 | 4 |
| 8.10 I have trouble buttoning buttons. | 0 | 1 | 2 | 3 | 4 |
| 8.11 I have trouble feeling the shape of small objects when they are in my hand. | 0 | 1 | 2 | 3 | 4 |
